# Assessment and Models of Insect Damage to Cones and Seeds of *Pinus strobiformis* in the Sierra Madre Occidental, Mexico

**DOI:** 10.3389/fpls.2021.628795

**Published:** 2021-04-29

**Authors:** Alejandro Leal-Sáenz, Kristen M. Waring, Rebeca Álvarez-Zagoya, José Ciro Hernández-Díaz, Carlos A. López-Sánchez, José Hugo Martínez-Guerrero, Christian Wehenkel

**Affiliations:** ^1^Programa Institucional de Doctorado en Ciencias Agropecuarias y Forestales, Universidad Juárez del Estado de Durango, Durango, Mexico; ^2^School of Forestry, Northern Arizona University, Flagstaff, AZ, United States; ^3^Instituto Politécnico Nacional, Durango, Mexico; ^4^Instituto de Silvicultura e Industria de la Madera, Universidad Juárez del Estado de Durango, Durango, Mexico; ^5^SMartForest Group, Department of Biology of Organisms and Systems, Mieres Polytechnic School, University of Oviedo, Mieres, Spain; ^6^Facultad de Medicina Veterinaria y Zootecnia, Universidad Juárez del Estado de Durango, Durango, Mexico

**Keywords:** X-ray, stereomicroscopic analysis, seed production, machine learning, regression analyses

## Abstract

Insect damage to cones and seeds has a strong impact on the regeneration of conifer forest ecosystems, with broader implications for ecological and economic services. Lack of control of insect populations can lead to important economic and environmental losses. *Pinus strobiformis* is the most widespread of the white pines in Mexico and is widely distributed throughout the mountains of northern Mexico. Relatively few studies have examined insect damage to the cones and seeds of these pines, especially in Mexico. In this study, we therefore analyzed insect damage to cones and seeds of *P. strobiformis* in Mexico by using X-ray and stereomicroscopic analysis. The specific objectives of the study were (a) to characterize insect damage by measuring external and internal cone traits, (b) to assess the health of seeds and cones of *P. strobiformis* in the Sierra Madre Occidental, Mexico, and (c) to estimate the relative importance of the effects of different environmental variables on cone and seed damage caused by insects. We found that 80% of *P. strobiformis* seeds and 100% of the tree populations studied had damage caused by insects. Most seeds were affected by *Leptoglossus occidentalis, Tetyra bipunctata, Megastigmus albifrons*, and the Lepidoptera complex (which includes *Apolychrosis synchysis, Cydia latisigna, Eucosma bobana*, and *Dioryctria abietivorella*). The cones of all tree populations were affected by some type of insect damage, with Lepidoptera causing most of the damage (72%), followed by *Conophthorus ponderosae* (15%), the hemipteran *L. occidentalis* (7%), and the wasp *M. albifrons* (6%). The proportion of incomplete seeds in *P. strobiformis* at the tree level, cone damage by *M. albifrons* and seed damage in *L. occidentalis* were associated with various climate and soil variables and with crown dieback. Thus, cone and seed insect damage can be severe and potentially impact seed production in *P. strobiformis* and the reforestation potential of the species. The study findings will enable managers to better identify insects that cause damage to cone and seeds. In addition, identification of factors associated with damage may be useful for predicting the levels of insect predation on seeds and cones.

## Introduction

Insect damage to tree cones and seeds is an important factor (Bramlett et al., 1977; Hedlin et al., 1980) affecting the regeneration of conifer forest ecosystems and also has broader implications for ecological and economic services (Wickman, 1992). Large economic and environmental losses can be generated if insect populations are not adequately controlled (Ruth et al., 1982; Dormont et al., 1996).

Multiple insect orders affect both the cones and seeds of several Mexican pines (Hedlin et al., 1980; Cibrián-Tovar et al., 1986); previous studies identified 49 insect species, belonging to six orders and 11 families, which attack cones and seeds of conifers in Mexico (Del Río-Mora, 1980; Hedlin et al., 1980; Cibrián-Tovar et al., 1986, 1995). Cone infestation and damage can reach high levels in natural forests. According to Blake et al. (1989), mortality rates reached between 38 and 81% in Arizonan *Pinus ponderosa* Doug. cones in 1984, as a result of damage caused by *Dioryctria auranticella* Grote (Lepidoptera: Pyralidae); damage by *Conophthorus ponderosae* Hopkins (Coleoptera: Scolytidae) also provoked cone mortality of between 3.9 and 36.4 %, while *Megastigmus albifrons* Walker (Hymenoptera: Torymidae) caused death of between 46 and 70% of seeds.

The insects known to damage the cones and seeds of Mexican conifers belong to seven orders, Coleoptera: represented by cone beetles in the genus *Conophthorus* spp. (Curculionidae: Scolytinae) are widely distributed and highly destructive to Mexican pine seed, and the cone weevil, *Conotrachelus neomexicanus* Fall. (Curculionidae: Molitinae) (Cibrián-Tovar et al., 1986). The order Hemiptera: include other relevant and harmful pests such as the western conifer seed bug, *Leptoglossus occidentalis* Heidemann (Hemiptera: Coreidae), and the shieldbacked pine seed bug, *Tetyra bipunctata*, Herrich-Schäffer (Hemiptera: Pentatomidae). Other pests of importance include the Hymenoptera: seed wasps, *M. albifrons* Walker (Torymidae). The Lepidoptera: such as cone borers, *Dioryctria, Eucosma*, and *Cydia*, is a genera with relevant species to cones of most of the Mexican pines. In the order Diptera: the southern cone gall midge, *Cecidomyia bisetosa* Gagné and the cone resin midge, *Contarinia* spp. and *Asynapta* spp. Felt (both, Cecidomyiidae), are important species that cause damage to cones. Homoptera: *Chionaspis pinifoliae* Fitch may become a pest of relative relevance for Mexican pines, covering some part of developed cones. Thysanoptera: has some species, such as *Haplothrips* spp. (Phlaeothripidae) that causes damage to conelets (Hedlin et al., 1980; Ruth et al., 1982; Cibrián-Tovar et al., 1986; Álvarez-Zagoya and Márquez-Linares, 1994a,b; Fairweather et al., 2006; Salinas-Moreno et al., 2010; Bustamante-García et al., 2012; DePinte et al., 2020).

*T. bipunctata* causes partial damage to second year seeds. But *L. occidentalis* leaves empty or almost totally empty seeds in second year cones (Hedlin et al., 1980; Cibrián-Tovar et al., 1986). DeBarr (1970) and Bramlett et al. (1977) reported that the type and size of seed damage depend on the size of the oral apparatus of the seedbugs, since there is a greater or lesser penetration through the layers of the scales. In *L. occidentalis*, if feeding occurred during the second year of cone development, a small feeding scar is evident on the mature seed covers (DePinte et al., 2020).

X-ray analysis is a non-destructive, rapid method that is commonly used to analyze conifer seed health (DeBarr, 1970; Bramlett et al., 1977; Chavagnat, 1985; Lippitt et al., 1994; Machado and Cícero, 2003; Himanen et al., 2015). It has been used to study several pine species, including *Pinus banksiana* Lamb., *Pinus elliottii* Engelm., *Pinus palustris* Mill., *Pinus ponderosa* Laws., *Pinus echinata* Mill., *Pinus taeda* L., *Pinus virginiana* Mill., *Pinus strobus* L., *Lithraea molleoides* (Vell.) Engl. and *Picea abies* L. Cone damage in pines is also assessed by other methods, like measurement of dissected cones scale by scale (Blake et al., 1989), cone measurements depending on sampling date, age and damage category (Bracalini et al., 2013), and determining the percentage of each type of damage in cones of *Pinus ponderosa* Douglas, *Pinus cembra* L., *Pinus albicaulis* Engelm, *Pinus pinea* and *P. strobiformis* Engelm. (Schmid et al., 1984; Dormont et al., 1996; Kegley et al., 2001; Bracalini et al., 2013; Linhart et al., 2014; DePinte et al., 2020).

Insect populations are strongly affected by deforestation and climate variation (Dunn and Crutchfield, 2006). High reproduction rates may not occur if occasional high temperatures prevent survival until maturity of insects in temperate and tropical regions (Kingsolver et al., 2011). Klapwijk et al. (2013) concluded that Lepidoptera species are positively affected by some changes in weather conditions (increased temperature and precipitation). In other species of insects, such as some members of the order Heteroptera, the increase in average summer temperature and decrease in the winter temperature can improve the potential conditions for hibernation and enable the species to extend to wider ranges, where they were not previously found (Musolin, 2007). In a study of the distribution of invasive insect populations in New Zealand, a high proportion of sites where some insects are present were found to be correctly classified by temperatures in late spring and early summer (Peacock et al., 2006). Moreover, several studies reported that certain soil conditions may be correlated with distribution, seasonal activities. and abundance of insects (e.g., McColloch and Hayes, 1922; Choudhuri and Banerjee, 1975) and insect attacks (Altieri and Nicholls, 2003).

Regression models are commonly used to predict probabilities of insect attacks and damage as a function of environmental factors and specific features of stands and individual trees (e.g., Negrón et al., 2009) and to harness scientific knowledge for effective ecosystem management (Seidl et al., 2011). Several comprehensive models of forest susceptibility to insect attack and damage were reported, such as by Luther et al. (1997) for *Acleris variana* Fern. and Wulder et al. (2006) for *Dendroctonus ponderosae* Hopk. particularly, however, machine learning algorithms demonstrated high capacity to predict physical condition of trees associated with insect damage, such as to forecast tree death or survival following the attack of *Thecodiplosis japonensis* Uch. et Inou where artificial neural networks (Park and Chung, 2006) were used to predict potential sanitary fellings of bark beetle-attacked Norway spruce, based on 21 climate, soil, and forest variables through multivariate regression trees (Ogris and Jurc, 2010) and also to estimate defoliation of Scots pine stands in western Poland, by *k*-nearest neighbors, random forest, and support vector machines as well as Sentinel-2 vegetation indices (Hawryło et al., 2018). White pines (genus *Pinus*, subgenus *Strobus*) are common tree species in temperate forest ecosystems. These species provide wood, resin, and pulp as well as valuable ecological services, such as provision of wildlife habitat (Farjon and Styles, 1997; Richardson, 2000; Villalobos-Arámbula et al., 2014). White pine seeds are large and nutritious and provide a valuable food source for rodents, parrots and other birds (Samano and Tomback, 2003; Looney and Waring, 2013). Conserving these pine species and their genetic diversity is therefore of great ecological importance (Bower et al., 2011). *P. strobiformis* is the most widespread of the white pines in Mexico and is widely distributed throughout the mountains of northern Mexico, from northern Chihuahua to the state of Jalisco in the south (Shirk et al., 2018; Leal-Sáenz et al., 2020).

*P. strobiformis* is currently facing multiple threats; e.g., range distribution models indicate that distribution of this species will contract in Mexico by up to 60% by 2080, due to increasingly unfavorable climate conditions (Shirk et al., 2018). The species is also highly susceptible to white pine blister rust, an invasive tree disease caused by the fungus *Cronartium ribicola* J. C. Fisch. (Geils et al., 2010), which occurs in the southwestern United States (not yet found in Mexico). Moreover, insect damage to *P. strobiformis* could exacerbate any future decline in the species (Looney and Waring, 2013; DePinte et al., 2020).

Relatively few studies have examined insect damage to cones and seeds of *P. strobiformis*, especially in Mexico (see Cibrián-Tovar et al., 1986, for general information and DePinte et al., 2020, for a similar study in the southwestern United States). In this study, we therefore analyzed insect damage to the cones and seed of *P. strobiformis* in Mexico. The specific study objectives were as follows: (a) to characterize insect damage based on external and internal cone traits; (b) to assess the health of seeds and cones of *P. strobiformis* in the Sierra Madre Occidental, Mexico; and (c) to estimate the relative importance of the effects of different environmental factors on cone and seed damage caused by insects. We expect the study findings to help us understand more about the environmental factors contributing to the probability of detecting particular insect species and also to enable us to estimate the damage in the sampled populations.

## Materials and Methods

### Species and Study Sites

**Figure 1:**
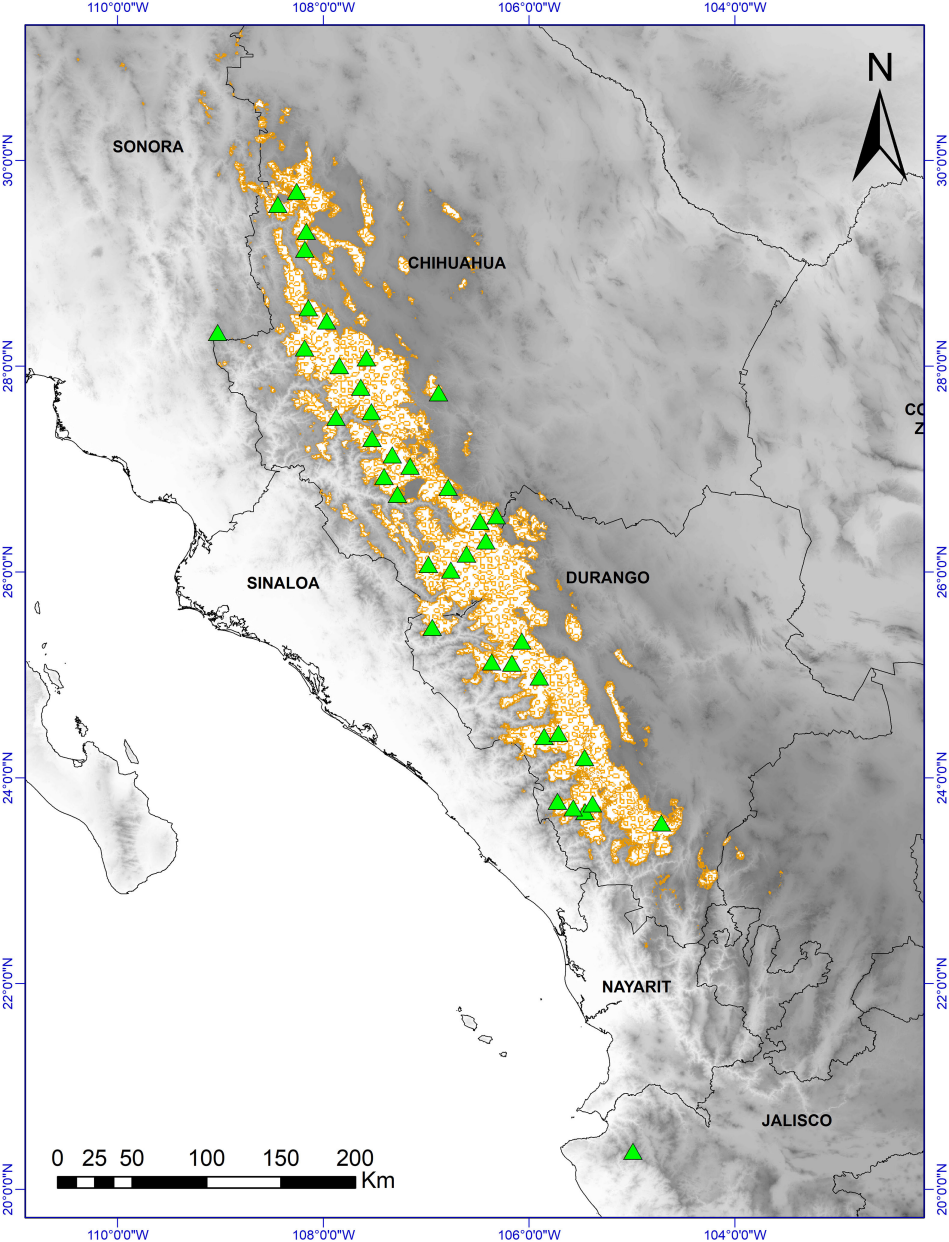
Distribution of *Pinus strobiformis* (brown outlined areas) and sample collection sites: populations (x40) (green triangles) in which 192 trees were sampled; distribution map based on Shirk et al. (2018). Base digital elevation map from Jarvis et al. (2008).

In Mexico, *P. strobiformis* is distributed in the state of Sonora, western Chihuahua and Durango, and northern Jalisco and Zacatecas (Figure 1; Looney and Waring, 2013; Villagómez-Loza et al., 2014). *P. strobiformis* mainly occurs at elevations of between 2,500 and 3,000 m, with a mean daily downwelling shortwave solar radiation of 15 kW m^−2^ (Aguirre-Gutiérrez et al., 2015).

In 2015, we collected cones and seeds from 40 *P. strobiformis* populations (192 trees) (Figure 1). The minimum distance between sites was 50 km, and individual trees were separated by a minimum distance of 50 m. The stands were located in closed forests, with minimal human disturbance. Three to five trees with mature cones were selected for study in each population, collecting at least 15 cones per tree (total 4,455 cones), which were also used for another study. The cones were randomly chosen from the upper third of the crown of each tree studied and all seeds were taken from these cones (Supplementary Table 1).

### Data Collection

#### Cone and Seed Damage by Insects

##### X-Ray Scanning of Seeds

In order to observe damage inside seeds, then, 10 seeds were randomly chosen per tree (i.e., total 1,920 seeds) and were X-rayed using Faxitrion X-Ray Corp. Model MX-20 2006 ® equipment. The X-ray was used to produce an image (tagged image file format, TIFF) of these seeds.

**Figure 2:**
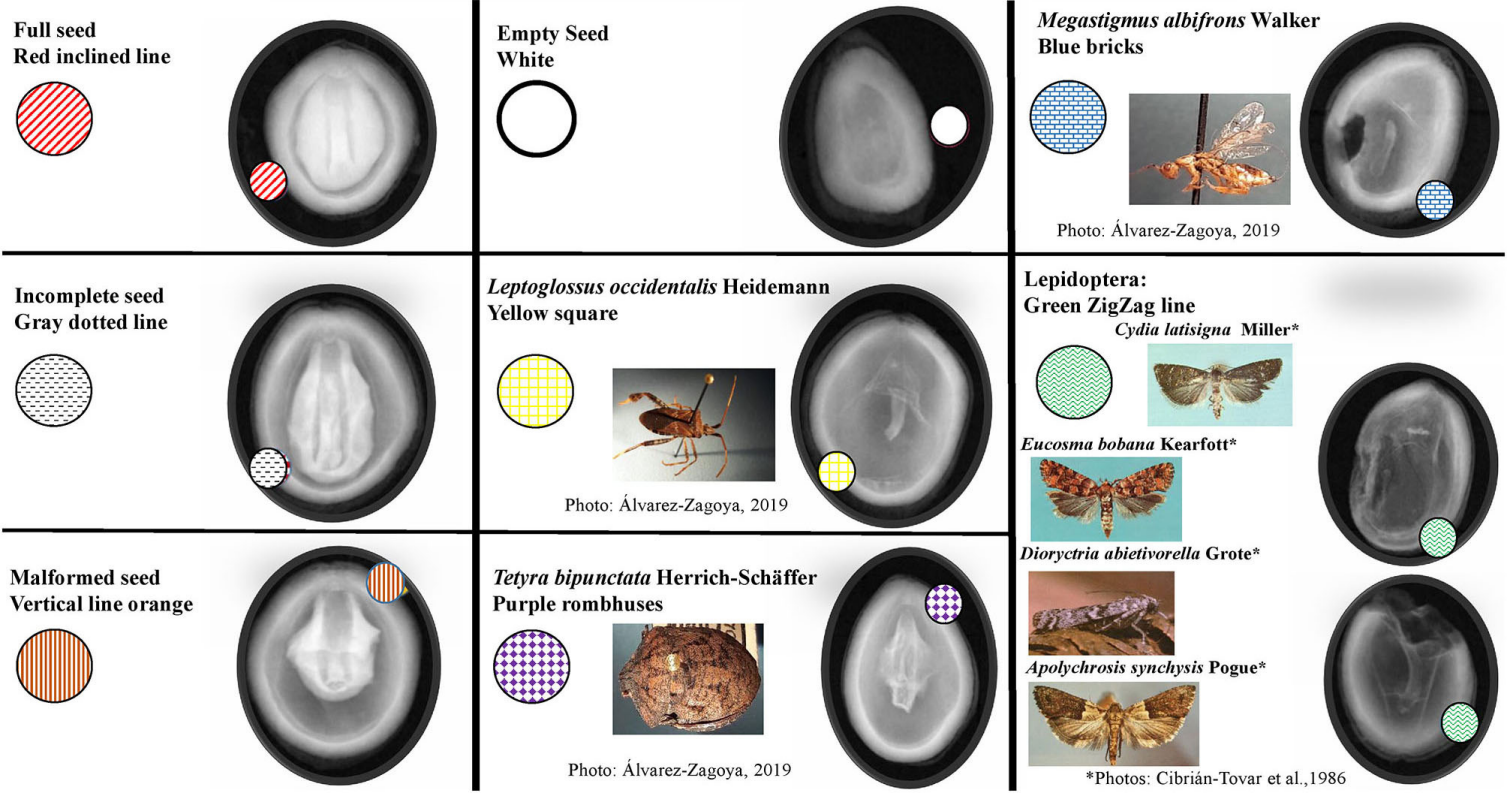
Identification guide to types of damage in *Pinus strobiformis* seeds.

All seeds were examined to evaluate whether they were affected by insect damage, and to identify the type of damage, according to the morphological characteristics of the insect and previously published reports (Bramlett et al., 1977; Hedlin et al., 1980; Ruth et al., 1982; Cibrián-Tovar et al., 1986; Álvarez-Zagoya and Márquez-Linares, 1998; Bustamante-García et al., 2012; DePinte et al., 2020). Damage severity was assigned using shape and color coding (Bustamante-García et al., 2012) in the IMAGE J program (Table 1). As the damage caused by the four species of Lepidoptera reported here was very similar, it was reported as a single factor (Table 1; Figure 2). Finally, we created an identification guide to insect damage on X-ray seeds.

**Table 1:** Color coding assigned by category of seed damage in Image-J.

**Seed characteristic and type of seed damage**	**Shape**
Full seed	Red dashed line
Incomplete seed	Gray dotted line
Malformed seed	Orange vertical line
Empty seed	White space
*Leptoglossus occidentalis* Heidemann	Yellow square
*Tetyra bipunctata* Herrich-Scháffer	Purple rhombuses
*Megastigmus albifrons* Walker	Blue rectangles
Lepidoptera group: *Cydia latisigna* Miller *Eucosma bobana* Kearfott *Dioryctria abietivorella* Grote *Apolychrosis synchysis* Pogue	Green zigzag line

##### Assessment of Cone Damage

Five cones per site (i.e., total 200 cones) were randomly chosen from the collected cones to identify insect damage to the outer and inner parts of the cone. One year after collection, the cones were cut lengthwise through the middle section, with a metal saw (Supplementary Table 1).

**Figure 3:**
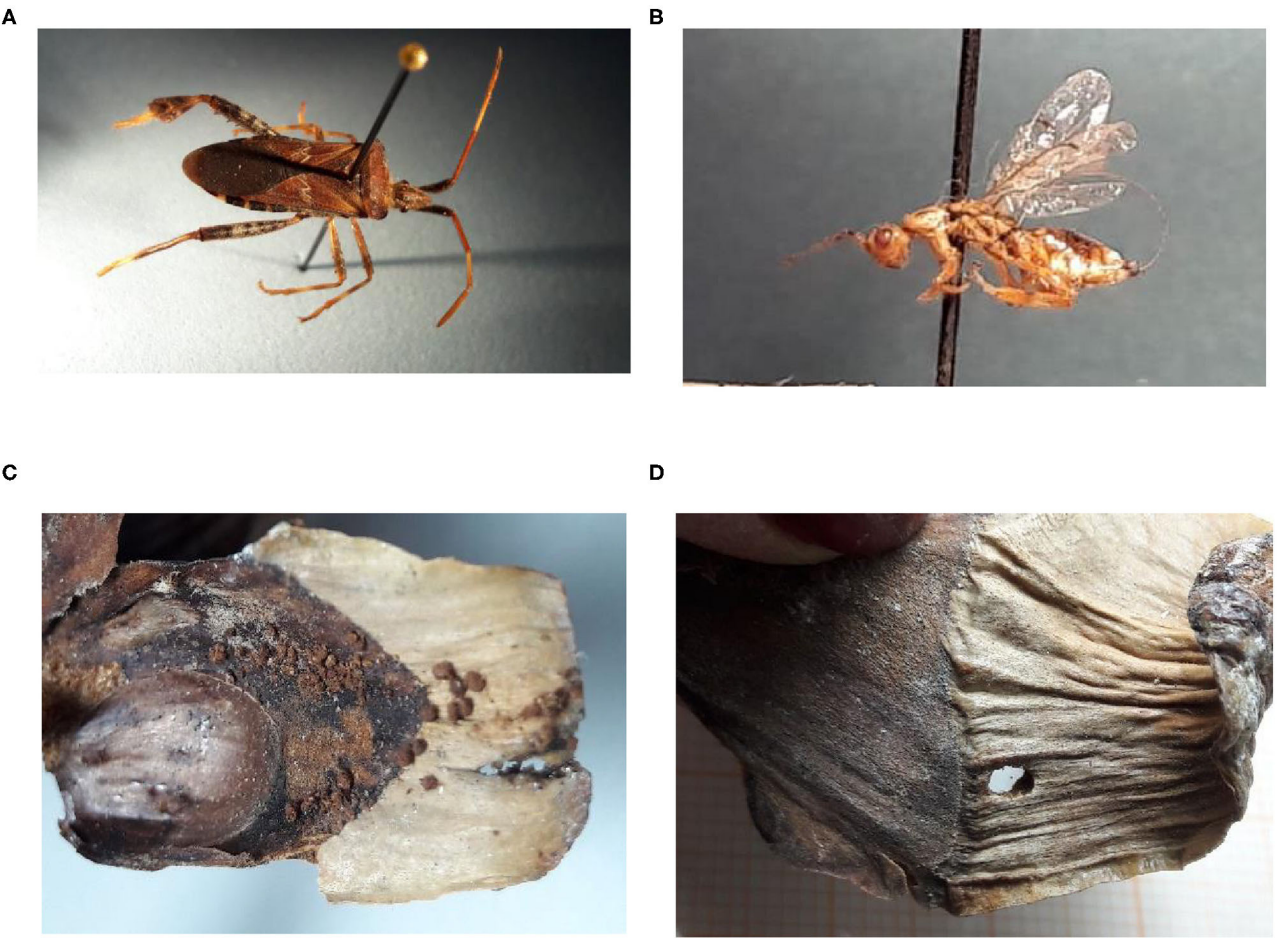
Examples of insects and the damage they cause in cones of *Pinus strobiformis*: **(A)** adult *Leptoglossus occidentalis* (Heidemann) (Photo: Álvarez-Zagoya, 2019), **(B)** adult *Megastigmus albifrons* (Walker) (Photo: Álvarez-Zagoya, 2019), **(C)** damage with frass by Lepidoptera [*Apolychrosis synchysis* (Pogue), *Cydia latisigna* (Miller), or *Eucosma bobana* (Kearfott), *Dioryctria abietivorella* (Grote)], **(D)** damage by *Conophthorus ponderosae* (Hopkins).

A Canon Power Shot A640 camera was used to capture an exterior image of each half of each cone, and each image was enlarged four times. Insect damage was then evaluated according to previous guidelines (Hedlin et al., 1980; Ruth et al., 1982; Cibrián-Tovar et al., 1986; Bustamante-García et al., 2012; DePinte et al., 2020). The damage was examined under a stereomicroscope (Zeiss stemi 2000C with an ocular magnification of 10X and objective magnification of 6X) (Figure 3).

#### Geographic, Topographic, and Dendrometric Data

For each sample tree, the latitude, longitude, elevation (m) and geographical aspect (0–360°) were recorded: for further analysis, the geographical aspect (0–360°), position of the slope (%), disease damage (dieback, shoot death, and leader condition), diameter at breast height (DBH, 1.3 m, recorded in cm), total tree height (m), height to the base of the live crown (m), and first live branch height (m) were determined (Table 2). The geographical aspect was transformed into a cosine index (as 0° and 360° have the same zenithal aspect) (Cushman and Wallin, 2002).

**Table 2:** Site characteristics and dasometric traits in a sample of 192 *Pinus strobiformis* trees.

**Variable**	**Minimum**	**Maximum**	**Mean**	**SD**
Slope (%)	0.3	91.6	26.9	19.5
Aspect (°)	1	129	25	25
Lat	20.371	29.700	26.457	1.917
Long	104.711	109.030	106.896	1.044
Elevation (m)	2,036	2,793	2,451	171
Dieback	0.0	20.0	4.0	5.2
Shoot death	0.0	4.0	0.4	0.8
Leader condition	0.0	8.0	0.6	1.4
**Dasometric trait**				
DBH (cm)	11.1	99.5	36.9	12.8
Height (m)	6.0	32.0	16.8	4.5
HBLC (m)	1.0	21.0	5.3	3.0
HBLLC (m)	0.1	18.0	4.6	2.7

*DBH, Diameter at breast height; Height, tree height; HBLC, height of the base of the live crown; HBLLC, first live branch height; SD, standard deviation; Aspect, geographical aspect; Lat, Latitude; Long, Longitude; Dieback, frequency of occurrence of dieback in the neighborhood; Shoot death, frequency of occurrence of shoot death in the neighborhood; leader condition, frequency of occurrence of leader condition in the neighborhood*.

#### Site Soil Analysis

In each of the 40 sites, a soil sample was obtained at a depth of 15 cm, which has been recommended in forest soil surveys manual (CONAFOR, 2011; Mendoza and Espinoza, 2017). Four soil subsamples (each 250 g) were collected at the base of four *P. strobiformis* trees studied and were mixed to make a composite sample of 1,000 g for each site.

In total, 27 soil variables were analyzed. Texture (relative proportions of sand, silt, and clay), density (Den) (g/cm^3^), concentration of calcium carbonate (CaCO_3_) (%), pH (CaCl_2_, 0.01 M), concentrations of potassium (K) (ppm), magnesium (Mg) (ppm), sodium (Na) (ppm), copper (Cu) (ppm), iron (Fe) (ppm), manganese (Mn) (ppm), zinc (Zn) (ppm), and calcium (Ca) (ppm) in the soil were determined by the methods described by Castellanos et al. (1999). Phosphorus (P) (ppm) was determined by the method of Olsen et al. (1954), while nitrate (NO_3_) (kg /ha) was determined by the method of Baker (1967), and the relative organic matter (OM) content (%) was determined by the method of León and Aguilar (1987). Electrical conductivity (CE) (dS/m) was determined by the method described by Vázquez and Bautista (1993). The cation exchange capacity (meq 100 g soil) (CEC) and the relative proportions (%) of hydrogen, Ca, M, K, Na, and other bases (o.b.) in the CEC were estimated on the basis of the Ammonium Acetate Method (pH 8.5) (Knudsen et al., 1982; Lanyon and Heald, 1982). The hydraulic conductivity (HC) (cm/h) was determined by the method of Mualem (1976), and percent saturation (Sat) (%) was estimated by the method of Herbert (1992). The soil analyses are summarized in Supplementary Table 2.

#### Occurrence of Woody Plants

At each site, we also recorded the following biotic factors: occurrence of the regeneration of *Pinus strobiformis*, presence of *Ribes* spp. (alternative host of white pine blister rust) and the presence of woody species in the neighborhood (*Cupressus, Pinus lumholtzii, Pinus arizonica, Pinus cooperi, Pinus durangensis, Pinus engelmannii, Pinus leiophylla, Pinus pseudostrobus, Pinus strobiformis, Pinus teocote, Quercus fulva, Quercus sideroxyla, Arctostaphylos pungens, Juniperus deppeana, Arbutus xalapensis, Castilleja angustifolia, Montanoa grandiflora, Filicopsida*). The presence of these species was recorded within a 20 m radius of each *P. strobiformis* sample tree. The occurrence data were transformed into frequency of occurrence prior to analysis (Supplementary Tables 3, 4). Climatic data (20 variables of temperature and precipitation) were downloaded from the PRISM database administered by the University of Idaho, USA (http://forest.moscowfsl.wsu.edu/climate/) (Supplementary Table 5).

### Data Analysis

To reach our objective of estimating the relative importance of the effects of different environmental factors (see Supplementary Table 5) on cone and seed damage, caused by the insects studied, we used statistical techniques to select those environmental independent factors that best predicted such damages and computed models for cone and seed damages based on those factors.

#### Feature Selection and Parametrization of Independent Environmental Factors Influencing Insect Damage

Adequate selection of significant independent variables influencing insect damage in the seeds and cones of 192 *P. strobiformis* trees was conducted by a Kruskal-Wallis test (KW), partial least squares (PLS) and the univariate curve of the receiving operator (ROC) (Krzanowski, 1987; Gnanadesikan et al., 1995; Maronna et al., 2018). The non-parametric Kruskal-Wallis test (Kruskal and Wallis, 1952) was applied to elevation and latitude data (Supplementary Tables 1–5).

The PLS and the univariate curve of the ROC with the “varImp” function was used along with the machine learning algorithm Random Forest (version 3.3.4; R Development Core Team, 2017). The weighted sums of the weights were determined with a function of the reduction in sum of squares with the PLS components (Kuhn, 2012).

The non-parametric Spearman correlation coefficient (*r*_*s*_) was used to determine collinearity between the significant independent variables of the 192 *P. strobiformis* trees, as indicated by the Kruskal-Wallis test, PLS or ROC. When the absolute value of the correlation coefficient between two variables was >0.70, only the variables with the lowest *p*-value were included in the models (as reported by Salas et al., 2017 and Shirk et al., 2018). Finally, all significant variables without collinearity (36 variables) from 78 mostly non-normally distributed independent variables (α = 0.01) were included in subsequent analyses.

#### Modeling Spatially Dependent Insect Damage Traits by Machine Learning Algorithms

To predict the most spatially dependent *P. strobiformis* cone and seed traits for selected important, independent variables of the 192 *P. strobiformis* sample trees, we used machine learning models, since they are fast, efficient and accurate for large-scale analyses (Badnakhe et al., 2018).

The number of variables for these models was determined by the rule of 10 events per variable (McGarigal et al., 2000; Vittinghoff and McCulloch, 2007: i.e., a maximum of the eight most important variables from the 192 trees was included in the models). These regression models, including five-fold cross-validations, were implemented in the “caret” package and function “train”: (i) linear regression (method = “lm”); (ii) Random forest (method = “rf”); (iii) Neural network (method = “nnet”); (iv) Average neural network model (method = “avNNet”); (v) Multilayer perceptron (method = “mlpWeightDecay”); and (vi) Bayesian Regularized Neural Networks (method = “brnn”) (Venables and Ripley, 1999; Williams et al., 2018, http://topepo.github.io/caret/index.html) in R (version 3.3.4) (R Development Core Team, 2017).

(Multiple) Linear regression is one of the simplest machine learning algorithms, often used for comparison purposes and creating parametric regression equations based on two (or more) parameters (Leal-Sáenz et al., 2020). A non-parametric Random Forest regression is based on constructing several decision trees at training time and outputting the average of the classes as the prediction of all individual trees (Breiman, 2001). A neural network is a series of algorithms intended to detect underlying relationships in a data set, mimicking the functioning of the human brain. A neural network comprises layers of interconnected model neurons (perceptrons) corresponding to multiple linear regressions. The model neuron sends the signal generated by multiple linear regression into an often non-linear activation function (see more in Krogh, 2008). We used Random Forests and Neural Networks because they have previously been applied in analysis of forest damages, and they have been found to perform better for events (especially in forestry) than other statistical methods (Hanewinkel, 2005; Guo et al., 2016). These seven algorithms were also successful to predict seed and cone traits of *P. strobifomis* by environmental variables (Leal-Sáenz et al., 2020).

The goodness of fit of the regression model was evaluated using the (pseudo) coefficient of determination (*R*^2^), the root mean square error (RMSE), and the mean squared error (MSE).

## Results

### Assessment of Seed and Cone Damage

We designed an identification guide that includes different categories of seed damage in *P. strobiformis*: incomplete, empty and malformed seeds, as well as damage caused by several types of insect (Figure 2). Representative damage by *L. occidentalis* and the Lepidoptera complex can be observed on the outside and inside of the seed. In *M. albifrons* and *T. bipunctata*, the damage is internal only.

Another identification guide for evaluating insect damage in cones of *P. strobiformis* was created using the data and images from the sites analyzed. Examples of cone damage by insects are described in the Figure 3. Excrement, silk threads, helical tunnels and reddish brown coloration in cones were typical types of frass produced by the Lepidoptera complex. On the other hand, *C. ponderosae* entered the subcortical cut and dug a tunnel along the axis of the cone, leaving a hole of approximately 2 mm at the entrance; the attack occurred in the basal third of the cone.

**Figure 4:**
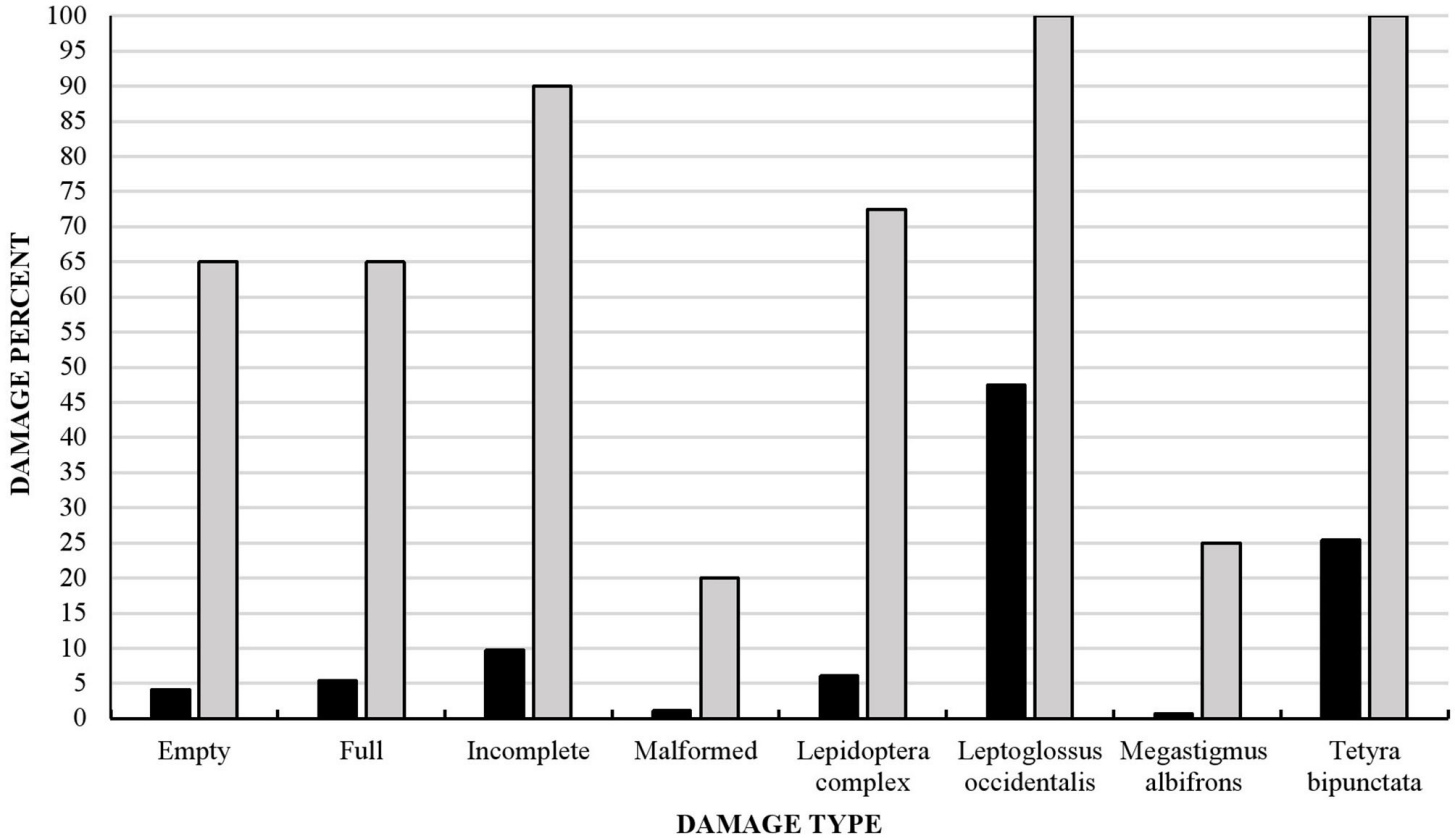
Insect damage in 1,920 seeds from 40 *Pinus strobiformis* populations sampled and studied at the seed (black) and insect population level (gray).

We found that 100% of the populations studied and 80% of *P. strobiformis* seeds had been damaged by insects (Figure 4). Most seeds were affected by the Lepidoptera complex as well as *M. albifrons, L. occidentalis*, and *T. bipunctata*. Although the last two species were found in all the populations studied, *M. albifrons* was only detected in 25% of the populations and the Lepidoptera complex in 73%. Only 20% of seeds in all the populations studied were undamaged or showed some other type of damage, not caused by insects (Figure 4).

**Figure 5:**
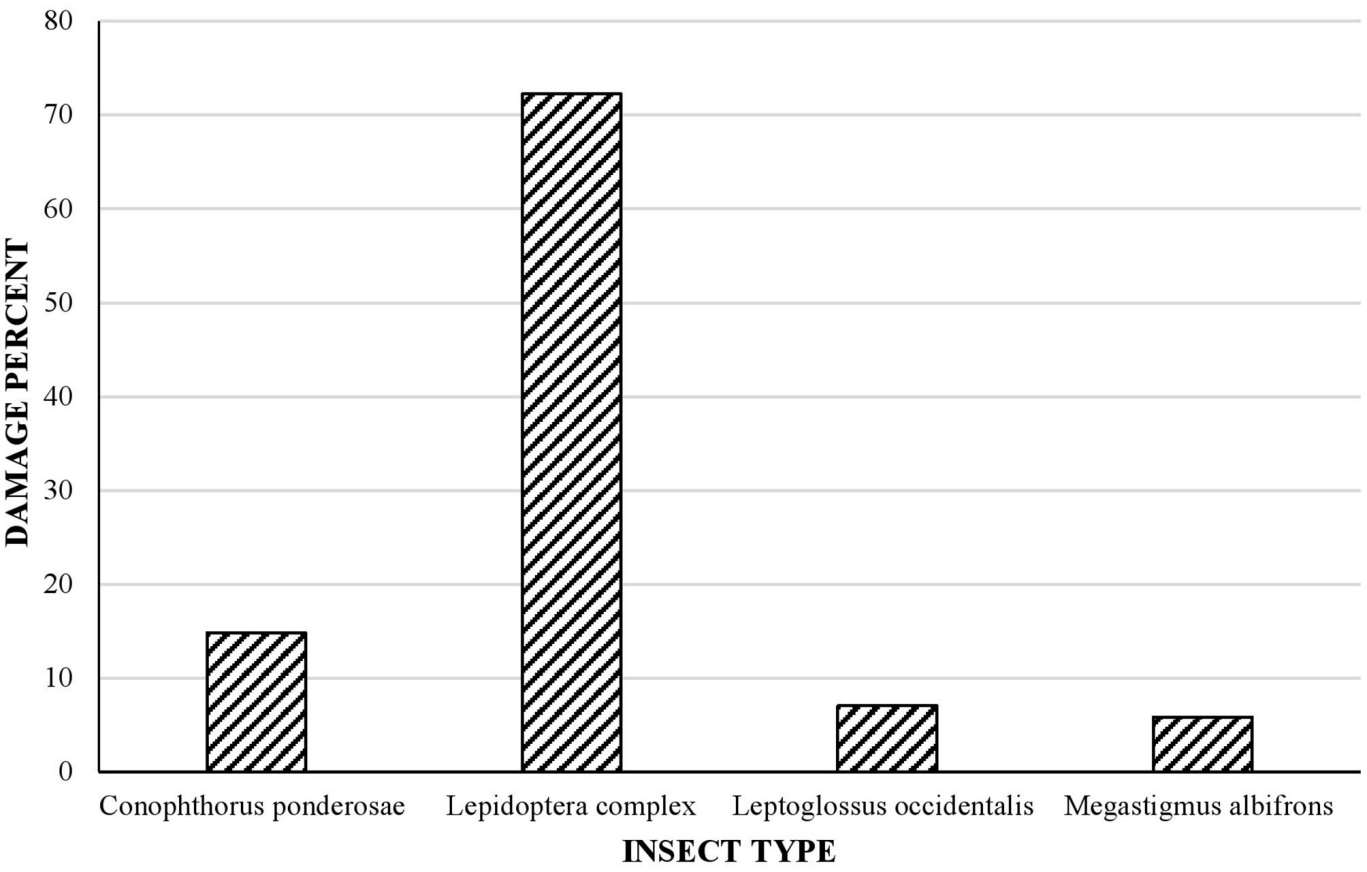
Percentage of cones damaged by different types of insect, determined in five randomly chosen cones per tree population studied (40 populations, total 200 cones). Note that some cones were damaged by more than one insect species.

All tree populations had some type of cone damage caused by members of the Lepidoptera complex, which was the most representative type (72% of damage) (Figure 5), followed by *C. ponderosae* (15% of damage), the hemipteran *L. occidentalis* (7% of damage), and the wasp *M. albifrons* (6% of damage).

### Modeling Seed and Cone Traits and Insect Damage in Relation to Environmental Variables by Using Regression Machine Learning Algorithms

The independent variables that together produced the best model to estimate incomplete seeds in *P. strobiformis* at tree level were the degree-days >5°C (DD5, degree-days), growing season precipitation (GSP, mm), winter precipitation (WINP, mm), summer precipitation balance (SMRPB, mm), manganese concentration in the soil (Mn, ppm), crown dieback (a measure of tree health), potassium concentration in the soil (K, ppm), and the proportion of Mg in CEC (Mg_CEC_), yielding an RMSE of 0.115 (proportion of damage) using the Bayesian Regularized Neural Networks (brnn) (Table 3; Supplementary Figure 1). The second-best model for estimating incomplete seed at tree level [RMSE = 0.118 (damage proportion)] was produced by the neural network (nnet) and included the following variables: mean temperature in the warmest month (MTWM, degrees C), SMRPB, dieback, GSP, percent saturation in the soil (%Sat), zinc concentration in the soil (Zn, ppm), Mn, and length of the frost-free period (FFP).

**Table 3:** Best fit models for estimating the percentage of “incomplete seed” damage, based on the sample of 192 *Pinus strobiformis* trees.

**Variable selection method**	**Machine learning algorithm**	**Independent variables**	**RMSE**	**MAE**	***R*^**2**^**
ROC	brnn	DD5, GSP, WINP, SMRPB, Mn_ppm_, dieback, K_ppm_, Mg_CEC_	0.115	0.090	0.342
KW_L_	nnet	MTWM, SMRPB, dieback, GSP, %Sat, Zn_ppm_, Mn_ppm_, FFP	0.118	0.098	0.325
KW_E_	avNNet	MMAX, FDAY, SMRPB, SPRP, dieback, shoot death, Slope, Zn_ppm_	0.119	0.095	0.323
PLS	lm	MAT, MMAX, EC, MAP, SMRPB, *Pinus cooperi*, MMIN, Na_ppm_	0.121	0.094	0.326
KW_E_	rf	MMAX, FDAY, SMRPB, SPRP, dieback, shoot death, Slope, Zn_ppm_	0.125	0.096	0.264
PLS	mlpWeightDecay	MAT, MMAX, EC, MAP, SMRPB, *Pinus cooperi*, MMIN, Na_ppm_	0.141	0.110	0.022

*ROC, Receiver Operating Characteristic; KW_L_, Kruskal Wallis selected with latitude; KW_E_, Kruskal Wallis selected with elevation; PLS, Partial Least Squares; brnn, Bayesian Regularized Neural Networks; nnet, Neural Network; avNNet, Model Averaged Neural Network; lm, linear regression; rf, Random Forest; mlpWeightDecay, Multi-Layer Perceptron; RMSE, Root-mean-square error; MAE, Mean Absolute Error; R^2^, Determination coefficient; DD5, Degree-days >5 degrees C (based on mean monthly temperature); GSP, Growing season precipitation (April to September); WINP, Winter precipitation: (nov+dec+jan+feb); SMRPB, Summer precipitation balance: (jul+aug+sep)/(apr+may+jun); Mn_ppm_, manganese concentration in the soil (ppm); dieback,Cronartium ribicola damage measurement; K_ppm_, potassium concentration in the soil (ppm); Mg_CEC_, proportion of magnesium in CEC (cation exchange capacity); MTWM, Mean temperature in the warmest month (degrees C); %Sat, Percent of water saturation; Zn_ppm_, zinc concentration in the soil (ppm); FFP, Length of the frost-free period (days); MAT, Mean annual temperature (degrees C); MMAX, Mean maximum temperature in the warmest month (degrees C); EC, Electrical conductivity (dS/m); MAP, Mean annual precipitation (mm); Pinus cooperi, frequency of occurrence of Pinus cooperi in the neighborhood; MMIN, Mean minimum temperature in the coldest month (degrees C); Na_ppm_, sodium concentration in the soil (ppm); FDAY, Julian date of the first freezing date of autumn; SPRP, Spring precipitation: (apr+may); shoot death, Cronartium ribicola damage measurement; Slope, slope position (%)*.

By contrast, MTWM and the regeneration of *P. strobiformis*, as well as several soil variables, including OM content (%), Zn, Mg in CEC, Cu concentration (ppm), and Hydraulic conductivity (HC, cm/h), were the variables that together provided the best prediction of the presence of cone damage in *P. strobiformis* by *M. albifrons* at population level (RMSE = 0.009) by using the Bayesian Regularized Neural Networks (brnn). The second best model for estimating the presence of cone damage percentage by the same insect (with RMSE = 0.010) was produced using the same variables and Random forest (rf) (Table 4; Supplementary Figure 2).

**Table 4:** Best fit models for estimating the percentage of cones damaged by *Megastigmus albifrons*, based on the sample of 192 *Pinus strobiformis* trees.

**Method of variable selection**	**Machine learning algorithm**	**Independent variables**	**RMSE**	**MAE**	***R*^**2**^**
PLS	brnn	%Organic matter, Zn_ppm_, K_CEC_, MTWM, Reg *P. strobiformis*, Cu_ppm_, Mn_ppm_, HC	0.0085	0.0061	0.983
PLS	rf	%Organic matter, Zn_ppm_, K_CEC_, MTWM, Reg *P. strobiformis*, Cu_ppm_, Mn_ppm_, HC	0.0100	0.0061	0.993
PLS	avNNet	%Organic matter, Zn_ppm_, K_CEC_, MTWM, Reg *P. strobiformis*, Cu_ppm_, Mn_ppm_, HC	0.0800	0.0691	0.454
PLS	nnet	%Organic matter, Zn_ppm_, K_CEC_, MTWM, Reg *P. strobiformis*, Cu_ppm_, Mn_ppm_, HC	0.0805	0.0693	0.462
PLS	mlpWeightDecay	%Organic matter, Zn_ppm_, K_CEC_, MTWM, Reg *P. strobiformis*, Cu_ppm_, Mn_ppm_, HC	0.0833	0.059	0.439
ROC	lm	CEC, MAP, Reg *P. strobiformis*, Mn_ppm_, MMAX, %Organic matter, SMRPB, Mg_CEC_	0.0834	0.0689	0.396

*PLS, Partial Least Squares; ROC, Receiver Operating Characteristic; brnn, Bayesian Regularized Neural Networks; rf, Random Forest; avNNet, Model Averaged Neural Network; nnet, Neural Network; mlpWeightDecay, Multi-Layer Perceptron; lm, linear regression; RMSE, Root-mean-square error; MAE, Mean Absolute Error; R^2^, Determination coefficient; %Organic matter, Relative proportion of organic matter in CEC (%) in the soil; Zn_ppm_, zinc concentration in the soil (ppm); K_CEC_, proportion of potassium in CEC (cation exchange capacity); MTWM, Mean temperature in the warmest month (degrees C); Reg P. strobiformis, Pinus strobiformis regeneration; Cu_ppm_, copper concentration in the soil (ppm); Mn_ppm_, manganese concentration in the soil (ppm); HC, Hydraulic conductivity (cm/h); CEC, Cation exchange capacity; MAP, Mean annual precipitation (mm); MMAX, Mean maximum temperature in the warmest month (degrees C); SMRPB, Summer precipitation balance: (jul+aug+sep)/(apr+may+jun); Mg_CEC_, proportion of magnesium in CEC (cation exchange capacity)*.

By contrast, SMRPB, DD5, WINP, Mn in the soil, K_CEC_, GSP, and the frequency of occurrence of *Arbutus xalapensis* and *Juniperus deppeana* in the neighborhood together provided the best prediction of seeds damaged in *P. strobiformis* by *L. occidentalis* at the tree level [with an RMSE of 0.199 (damage proportion) with the Model Averaged Neural Network (avNNet)]. The second best model of seed damage by *L. occidentalis* at tree level [RMSE = 0.201 (damage proportion)] was produced using the same variables and the Bayesian Regularized Neural Networks (brnn) approach (Table 5; Supplementary Figure 3).

**Table 5:** Best fit models for estimating percentage of seeds damaged by *Leptoglossus occidentalis*, based on the sample of 192 *Pinus strobiformis* trees.

**Variable selection method**	**Machine learning algorithm**	**Independent variables**	**RMSE**	**MAE**	***R*^**2**^**
ROC	avNNet	SMRPB, DD5, WINP, Mn_ppm_, K_CEC_, GSP, *Arbutus xalapensis, Juniperus deppeana*	0.199	0.162	0.122
ROC	brnn	SMRPB, DD5, WINP, Mn_ppm_, K_CEC_, GSP, *Arbutus xalapensis, Juniperus deppeana*	0.201	0.160	0.107
KW_E_	rf	MMAX, FDAY, SMRPB, SPRP, dieback, shootdeath, Slope, Zn_ppm_	0.203	0.163	0.140
KW_E_	nnet	MMAX, FDAY, SMRPB, SPRP, dieback, shootdeath, Slope, Zn_ppm_	0.203	0.164	0.076
ROC	lm	SMRPB, DD5, WINP, Mn_ppm_, K_CEC_, GSP, *Arbutus xalapensis, Juniperus deppeana*	0.204	0.165	0.111
KW_E_	mlpWeightDecay	MMAX, FDAY, SMRPB, SPRP, dieback, shoot death, Slope, Zn_ppm_	0.219	0.174	0.082

*ROC, Receiver Operating Characteristic; KW_E_, Kruskal Wallis selected with elevation; avNNet, Model Averaged Neural Network; brnn, Bayesian Regularized Neural Networks; rf, Random Forest; nnet, Neural Network; lm, linear regression; mlpWeightDecay, Multi-Layer Perceptron; RMSE, Root-mean-squared error; MAE, Mean Absolute Error; R^2^, Determination coefficient; SMRPB, Summer precipitation balance: (jul+aug+sep)/(apr+may+jun); DD5, Degree-days >5 degrees C (based on mean monthly temperature); WINP, Winter precipitation: (nov+dec+jan+feb); Mn_ppm_, manganese concentration in the soil (ppm); K_CEC_, proportion of potassium in CEC (cation exchange capacity); GSP, Growing season precipitation (April to September); Arbutus xalapensis, frequency of occurrence of Arbutus xalapensis in the neighborhood; Juniperus deppeana, frequency of occurrence of Juniperus deppeana in the neighborhood; MMAX, Mean maximum temperature in the warmest month (degrees C); FDAY, Julian date of the first freezing date of autumn; SPRP, Spring precipitation: (apr+may), dieback, Cronartium ribicola damage measurement; shoot death, Cronartium ribicola damage measurement; Slope, slope position (%); Zn_ppm_, zinc concentration in the soil (ppm)*.

The SMRPB was negatively correlated with the percentage of incomplete seeds (Supplementary Table 6). The percentage of cones damaged by *M. albifrons* was positively correlated with the OM content (%) of the soil and negatively correlated with the regeneration of *P. strobiformis* and Mn in the soil (Supplementary Table 7). Seed damage by *L. occidentalis* was positively correlated with SMRPB, DD5, WINP, Mn, K_CEC_ in CEC and negatively correlated with GSP and the frequency of occurrence of *A. xalapensis* and *J. deppeana* in the neighborhood (Supplementary Table 8).

## Discussion

### Characterization of Insect Damage

We found a wide variety of insect damage in seeds and cones of the Mexican *P. strobiformis* populations studied, reported here for the first time. Our guide to identification of the seed and cone damage will help managers to characterize damage, identify causal agents and will also help in monitoring seed and cone damage by insects. Identifying the damaging agent is important as insect abundance and distribution are likely to vary across the range of *P. strobiformis* (DePinte, 2016). Identifying the insects that cause the damage, the locations involved and the proportion of the cone and seed production for a given location is critical for predicting available seed for reforestation and proactive management. While seed and cone insects comprise one of the least understood insect guilds in regard to climate change interactions, the relationships between insects and hosts will probably change (Pureswaren et al., 2018). High levels of insect damage to cone and seed production could greatly reduce the regeneration potential and also the availability of healthy seeds for use in seedling production, research and tree breeding programs (Hedlin et al., 1980; Cibrián-Tovar et al., 1986).

Several of the damage indicators we report here have previously been found in other host species. Bracalini et al. (2013) reported resin excess as an indication of *L. occidentalis* attack, along with the interaction with several other insects present in cones and seeds of *P. pinea* in Italy. The Lepidoptera complex insect indicators, including agglomeration of frass, pupae, and a reddish coloration, reported in previous research (Hedlin et al., 1980; Cibrián-Tovar et al., 1986; Whitehouse et al., 2011), were also observed in the present study.

### Assessment of Seed and Cone Health

We observed a high percentage of insect damage in *P. strobiformis* seeds and cones at both seed and population levels. However, Kelly and Sork (2002) reported that cone and seed production are adaptive traits that are spatially and temporally variable, with several years of cone and seed production driven by both predation avoidance and pollen dispersal. Insect predators and their host trees have closely linked developmental stages, leading to fluctuations in insect populations that generally follow oscillations in cone and seed production (Turgeon et al., 1994; Poncet et al., 2009). Our results from a single year observations provide insight into the potential for damage but, indeed, additional research is needed to reveal long-term spatial and temporal patterns of insect abundance, distribution, and population dynamics and the consequent damage to *P. strobiformis* reproductive potential.

*Leptoglossus occidentalis* was found to cause the most damage in the present study, followed by *Tetyra bipunctata*. Previous research in *Pseudotsuga mensiezii* found that light to moderate feeding by *L. occidentalis* reduced seed germination by 80% (Bates et al., 2001). Kegley et al. (2001) showed that *Dioryctria abietivorella* (Grote) and *L. occidentalis* produced the highest level of damage in cones and seeds of another five-needle pine species, whitebark pine (*P. albicaulis* Engelm.). Schwandt et al. (2010) also reported that cone and seed insects can occasionally cause high losses of cones/seeds in five-needle pines. In the northern range of *P. strobiformis* in the United States, DePinte et al. (2020) observed damage by *D. abietivorella, Eupithecia spermaphaga, C. ponderosae*, and *L. occidentalis*. Similar to the range in Mexico, *L. occidentalis* was the most common damaging agent (DePinte, 2016). This insect caused damage in almost half of the sampled cones and seed in the present study, and it has the capacity to cause substantial damage. In Europe, *L. occidentalis* is an invasive species and has been found to cause damage in up to 70% of seeds in natural pine stands (Lesieur et al., 2014). Some other insects found to cause little damage in the present study (for example, *M. albifrons* damaged 1% of seeds and 6% of cones), also have the potential to cause higher levels of damage and affect reproductive output: Blake et al. (1989) reported a loss of 70% in cones of *Pinus ponderosa* Doug in northern Arizona, United States, caused by *M. albifrons*.

Five-needle pines in North America, such as *P. strobiformis*, are of special concern due to their susceptibility to the non-native invasive tree disease, white pine blister rust (caused by the fungal pathogen *C. ribicola*) (Geils et al., 2010). White pine blister rust has not yet been documented in Mexico, but occurs just north of the Mexican border in New Mexico (USA), on *P. strobiformis* (Conklin et al., 2009). Maintaining robust and healthy cone and seed production is critical for enhancing natural regeneration and providing seed for genetic resistance breeding efforts and artificial reforestation (Sniezko et al., 2018; Schoettle et al., 2019). Our findings indicate a need for increased monitoring of cone and seed insect populations and damage, particularly as climate change is increasing tree mortality in Mexico (Sáenz-Romero et al., 2020).

### Modeling Seed and Cone Traits and Insect Damage, Based on Environmental Variables Using Regression Machine Learning Algorithms

The percentage of incomplete seeds in *P. strobiformis* at the tree level was correlated with various climate and soil variables and with crown dieback. The most important variables were degree-days >5°C (DD5), GSP, WINP, and SMRPB (Table 3). In a study involving *Pinus sylvestris* var. *Mongolian*, He et al. (2018) found that the richness and divergence of the cone traits were positively correlated with water availability, while the seed diversity indices were negatively correlated with water availability. These researchers suggested that pines maximize the characteristics of their seeds in multiple dimensions to make their resources more efficient. Despland and Houle (1997) sampled *Pinus banksiana* serotinous cones produced between 1969 and 1992, observing that reproductive variables were positively associated with high temperatures, while we found that high temperatures were often associated with the percentage damage to incomplete seeds in this study (Table 3). Cain and Shelton (2000) reported that *P. taeda* seed production was positively correlated with the average monthly precipitation and negatively correlated with temperature.

We found that cone damage by *M. albifrons* was positively related to the OM content (%), zinc concentration and mean temperature in the warmest month (Table 4). Altieri and Nicholls (2003) also report that soils with a high content of OM and active biology manage better to prevent insect attack. Jamieson et al. (2012) suggest that heat and drought can cause plants to be less tolerant to insects. Several authors report the relationship between zinc and plant health; e.g., Broadley et al. (2007) and Di Baccio et al. (2005) mention that at certain concentrations zinc is toxic, but is an essential component of thousands of plant proteins. Zinc is the second transition metal after iron (Fe) and the only metal represented in the six enzyme classes (Broadley et al., 2007). Tsonev and Cebola Lidon (2012) showed that excess Zn^2+^ after a broad response to heavy metals (Cd, Mg, Cu) causes necrosis, wilt and a decrease in biomass; Tripathi et al. (2015) mentioned that Zn is a structural constituent or regulatory cofactor for different enzymes and proteins and that optimum culture growth is generally maintained by absorbing Zn in the divalent form. Zinc performs various important functions in plants: (i) it regulates carbonic anhydrase for binding to carbohydrates in plants; (ii) it promotes metabolism of carbohydrates, proteins, auxins, pollen formation; and (iii) it is an essential element in biological membranes and participates in defense mechanisms against harmful pathogens. Lee et al. (2007) concluded that one of the highest antioxidant defense mechanisms in transgenic tall fescue plants is the overexpression of the CuZnSOD and APX genes. Finally, Mihăiescu et al. (2011) and Wu et al. (2015) reported that in plants Zn generates greater resistance to hot and dry climates as well as resistance to bacterial and fungal diseases.

In the seed damaged by *L. occidentalis* the most important variables were SMRPB, degree-days >5°C (DD5), winter precipitation (WINP), and manganese concentration in the soil (Table 5). Zhu et al. (2014) reported that the north to south limits of distribution in America of *L. occidentalis* coincide with the limits of the coniferous host plants, which suggests that climate is the main limiting factor. According to Tripathi et al. (2015), manganese is an essential microelement whose bioavailability is affected by the pH (more than 6.5) of the soil and it participates in stress-tolerant mechanisms of higher plants by serving as a cofactor of various antioxidant enzymes. However, St. Clair and Lynch (2005) found that hyperaccumulation of Mn in tree species that grow in acidic soils negatively affects their health. Subrahmanyam and Rathore (2000) indicated that the Mn toxicity can reduce plant photosynthesis.

There is no direct information available about the impact of climate or nutrients on the development of pests in *P. strobiformis*, or other Mexican pines. The possible relationships or interactions of the attack by *M. albifrons* on the cones and by *L. occidentalis* on the seeds were therefore directly inferred from the data obtained.

## Conclusion

The information reported in this study is intended to enhance damage measurement parameters, both in the laboratory and in the field, to enable detection of insect damage in *P. strobiformis*. We confirmed that much of the damage to seeds caused by insects (80%) cannot be detected by visual observation and that analytical tools, such as X-ray techniques, are therefore required to reveal the full extent of the damage. Future research is needed to investigate spatial and temporal patterns of insect activity, population dynamics, and their relationships through time and space with environmental factors. The study findings contribute to predicting some types of insect damage and the proportion of viable seeds and could therefore be used to develop an integrated management programme for *P. strobiformis* pests.

## Data Availability Statement

The original contributions presented in the study are included in the article/Supplementary Material, further inquiries can be directed to the corresponding author/s.

## Author Contributions

AL-S and CW conceived and designed the experiments, conducted sampling, analyzed the data, and prepared figures and tables. AL-S and RÁ-Z identified the insect damages. KW and CW contributed reagents, materials, and analysis tools. CW and AL-S wrote the paper. All authors discussed the results, contributed to the article, and approved the submitted version.

## Conflict of Interest

The authors declare that the research was conducted in the absence of any commercial or financial relationships that could be construed as a potential conflict of interest.
