# Supplementary material for: Assessment and Models of Insect Damage to Cones and Seeds of Pinus strobiformis in the Sierra Madre Occidental, Mexico
Source: Front Plant Sci. 2021 Apr 29;12:628795. doi: 10.3389/fpls.2021.628795 (PMC8116514; doi:10.3389/fpls.2021.628795)
Supplement: Supplementary file 2 [file Data_Sheet_2.docx]

Supplementary Material

**Supplementary Table 1 |** Insect damage observed in cones and seeds in a sample of 192 *Pinus strobiformis* trees

| Variable | Minimum | Maximum | Mean | SD |
| --- | --- | --- | --- | --- |
| SEEDS |  |  |  |  |
| Full | 0 | 0.3 | 0.05 | 0.08 |
| Incomplete | 0 | 0.7 | 0.09 | 0.14 |
| Malformed | 0 | 0.8 | 0.01 | 0.06 |
| Empty | 0 | 0.8 | 0.04 | 0.10 |
| *Megastigmus albifrons* | 0 | 0.1 | 0.01 | 0.03 |
| *Tetyra bipunctata* | 0 | 0.7 | 0.25 | 0.16 |
| *Leptoglossus occidentalis* | 0 | 1 | 0.47 | 0.21 |
| Lepidoptera | 0 | 0.5 | 0.06 | 0.11 |
|  |  |  |  |  |
| CONES |  |  |  |  |
| *Leptoglossus occidentalis* | 0 | 0.8 | 0.08 | 0.17 |
| *Conophthorus ponderosae* | 0 | 0.8 | 0.18 | 0.19 |
| *Megastigmus albifrons* | 0 | 0.4 | 0.07 | 0.11 |
| Lepidoptera | 0.6 | 1 | 0.92 | 0.13 |

**Supplementary Table 2 |** Analysis of soil samples from 192 *Pinus strobiformis* trees.

| Variable | Minimum | Maximum | Mean | SD |
| --- | --- | --- | --- | --- |
| Electrical conductivity (dS/m) | 0.25 | 3.08 | 1.45 | 0.70 |
| Nitrate (kg/ha) | 29.57 | 1084.30 | 439.29 | 197.25 |
| Phosphorus (ppm) | 7.39 | 154.82 | 54.27 | 37.77 |
| Organic matter (%) | 1.65 | 28.92 | 7.37 | 5.16 |
| Percent saturation (%) | 6.00 | 144.00 | 67.31 | 24.57 |
| Sand (%) | 36.70 | 84.70 | 63.01 | 10.32 |
| Silt (%) | 1.28 | 43.28 | 22.78 | 8.62 |
| Clay (%) | 12.02 | 24.02 | 14.21 | 2.84 |
| Density (g/cm3) | 0.52 | 1.11 | 0.85 | 0.15 |
| pH | 3.46 | 6.21 | 5.18 | 0.64 |
| Calcium (ppm) | 666.00 | 6132.00 | 2324.25 | 1167.29 |
| Magnesium (ppm) | 102.00 | 1494.00 | 307.50 | 255.10 |
| Sodium (ppm) | 30.00 | 114.00 | 56.02 | 18.21 |
| Potassium (ppm) | 212.00 | 4107.00 | 691.65 | 700.69 |
| Iron (ppm) | 24.72 | 368.28 | 66.49 | 59.53 |
| Zinc (ppm) | 0.40 | 7.52 | 1.59 | 1.47 |
| Manganese (ppm) | 0.44 | 126.00 | 26.57 | 29.64 |
| Copper (ppm) | 0.04 | 0.68 | 0.26 | 0.15 |
| Rel. proportion of other bases in CEC (%) | 5.23 | 7.09 | 6.57 | 0.71 |
| Rel. proportion of H in CEC (%) | 11.85 | 53.10 | 27.76 | 9.38 |
| Rel. proportion of Ca in CEC (%) | 14.59 | 67.72 | 47.55 | 9.46 |
| Rel. proportion of Mg in CEC (%) | 2.96 | 27.49 | 9.98 | 4.15 |
| Rel. proportion of K in CEC (%) | 1.64 | 21.01 | 6.99 | 3.69 |
| Rel. proportion of Na in CEC (%) | 0.40 | 2.95 | 1.15 | 0.54 |
| Cation exchange capacity (meq/100 g soil) | 8.15 | 55.37 | 24.08 | 9.72 |
| Hydraulic conductivity (cm/h) | 6.15 | 50.21 | 19.58 | 10.97 |

**Supplementary Table 3 |** Frequency of occurrence of tree species in association with *Pinus strobiformis*, estimated from a sample of 192 *Pinus strobformis* trees in Mexico.

| *Variable = frequency of occurrence | Minimum | Maximum | Mean | SD |
| --- | --- | --- | --- | --- |
| *Pinus arizonica* | 0 | 1 | 0.56 | 0.49 |
| *Pinus strobiformis* | 0 | 1 | 0.31 | 0.46 |
| *Pinus durangensis* | 0 | 1 | 0.35 | 0.48 |
| *Quercus sideroxyla* | 0 | 1 | 0.33 | 0.47 |
| *Pinus cooperi* | 0 | 1 | 0.19 | 0.39 |
| *Pinus leiophylla* | 0 | 1 | 0.19 | 0.39 |
| *Pinus lumholtzii* | 0 | 1 | 0.18 | 0.39 |
| *Pinus teocote* | 0 | 1 | 0.15 | 0.36 |
| *Pinus engelmannii* | 0 | 1 | 0.13 | 0.34 |
| *Quercus fulva* | 0 | 1 | 0.10 | 0.31 |
| *Cupressus* | 0 | 1 | 0.05 | 0.22 |
| *Pinus pseudostrobus* | 0 | 1 | 0.03 | 0.16 |
| *Ribes spp.* | 0 | 1 | 0.19 | 0.39 |
| Regeneration of *P. strobiformis* | 0 | 1 | 0.91 | 0.29 |
| *Juniperus deppeana* | 0 | 1 | 0.46 | 0.50 |

* SD = standard deviation.

**Supplementary Table 4 |** Frequency of occurrence of shrub species in association with *Pinus strobiformis*, estimated from a sample of 192 *Pinus strobformis* trees in Mexico.

| *Variable = frequency of occurrence of | Minimum | Maximum | Mean | SD |
| --- | --- | --- | --- | --- |
| *Montanoa grandiflora* | 0 | 1 | 0.02 | 0.12 |
| *Filicopsida* spp. | 0 | 1 | 0.09 | 0.29 |
| *Castilleja angustifolia* | 0 | 1 | 0.02 | 0.12 |
| *Arbutus xalapensis* | 0 | 1 | 0.39 | 0.48 |
| *Arctostaphylos pungens* | 0 | 1 | 0.56 | 0.49 |

* . SD = standard deviation.

**Supplementary Table 5 |** Climate characteristics determined in a sample of 192 *Pinus strobiformis* trees in Mexico (University of Idaho, USA (<http://forest.moscowfsl.wsu.edu/climate/)>).

| Variable | Min | Max | Mean | SD |
| --- | --- | --- | --- | --- |
| Mean annual temperature (degrees C) (MAT) | 9.2 | 13.1 | 10.9 | 0.9 |
| Mean annual precipitation (mm) (MAP) | 584 | 1817 | 942 | 218 |
| Growing season precipitation, April to September (GSP) | 457 | 1439 | 685 | 163 |
| Mean temperature (degrees C) in the coldest month (MTCM) | 3.3 | 10.0 | 5.1 | 1.2 |
| Mean minimum temperature (degrees C) in the coldest month (MMIN) | -6.5 | 3.0 | -4.3 | 1.5 |
| Mean temperature (degrees C) in the warmest month (MTWM) | 13.6 | 19.1 | 16.3 | 1.6 |
| Mean maximum temperature in the warmest month (MMAX) | 21.5 | 28.2 | 25.3 | 1.8 |
| Julian date of the last freezing date of spring (SDAY) | 77 | 171 | 150 | 13 |
| Julian date of the first freezing date of autumn (FDAY) | 254 | 332 | 281 | 12 |
| Length of the frost-free period (days) (FFP) | 88 | 251 | 136 | 25 |
| Degree-days >5 degrees C (based on mean monthly temperature) (DD5) | 1,782 | 2,961 | 2,326 | 299 |
| Degree-days >5 degrees C accumulating within the frost-free period (GSDD5) | 759 | 2,346 | 1,382 | 336 |
| Julian date the sum of degree-days >5 degrees C reaches 100 (D100) | 19 | 76. | 55 | 11 |
| Degree-days <0 degrees C (based on mean monthly temperature) (DD0) | 0 | 56 | 21 | 15 |
| Degree-days <0 degrees C (based on mean minimum monthly temperature) (MMINDD0) | 78 | 1,233 | 813 | 191 |
| Summer precipitation balance: (jul+aug+sep)/(apr+may+jun) (SMRPB) | 3.41 | 7.39 | 4.76 | 0.84 |
| ((Depreciated) Summer/Spring precipitation balance: (jul+aug)/(apr+may) (SMRSPRPB) | 10.10 | 21.07 | 13.25 | 2.06 |
| Spring precipitation: (apr+may) (SPRP) | 26 | 46 | 31 | 5 |
| Summer precipitation: (jul+aug) (SMRP) | 276 | 775 | 420 | 87 |
| Winter precipitation: (nov+dec+jan+feb) (WINP) | 82 | 333 | 178 | 49 |

SD = standard deviation.

**Supplementary Table 6 |** Parameters found for the best linear regression model of incomplete seed based on a sample of 192 *Pinus strobiformis* trees.

| (Intercept) | -0.0230 |
| --- | --- |
| MAT = Mean annual temperature (degrees C) | 0.0684 |
| MMAX = Mean maximum temperature in the warmest month (degrees C) | -0.0459 |
| EC = Electrical conductivity (dS/m) | 0.0486 |
| MAP = Mean annual precipitation (mm) | 0.0003 |
| SMRPB = Summer precipitation balance: (jul+aug+sep)/(apr+may+jun), | -0.0033 |
| *Pinus cooperi =* frequency of occurrence of *Pinus cooperi* in the neighborhood | -0.0429 |
| MMIN = Mean minimum temperature (degrees C) in the coldest month | -0.0621 |
| Na_ppm_ = sodium concentration in the soil (ppm) | -0.0009 |

**Supplementary Table 7 |** Parameters included in the best linear regression model of *Megastigmus albifrons* cone damage, based on a sample of 192 *Pinus strobiformis* trees.

| (Intercept) | 0.492 |
| --- | --- |
| CEC = Cation exchange capacity | -0.001 |
| MAP = Mean annual precipitation (mm) | -0.00005 |
| Reg *P. strobiformis = Pinus strobiformis* regeneration | -0.1431 |
| Mn_ppm_ = manganese concentration in the soil (ppm) | -0.0002 |
| MMAX = Mean maximum temperature in the warmest month (degrees C) | -0.0132 |
| %Organic matter = Relative proportion of organic matter in CEC (%) in the soil | 0.0076 |
| SMRPB = Summer precipitation balance: (jul+aug+sep)/(apr+may+jun) | 0.0217 |
| Mg_CEC_ = proportion of magnesium in CEC (cation exchange capacity) | -0.0033 |

**Supplementary Table 8 |** Parameters included in the best linear regression model of *Leptoglossus occidentalis* seed damage based on a sample of 192 *Pinus strobiformis* trees.

| (Intercept) | 0.303 |
| --- | --- |
| SMRPB = Summer precipitation balance: (jul+aug+sep)/(apr+may+jun) | 0.022 |
| DD5 = Degree-days >5 degrees C (based on mean monthly temperature) | 0.00009 |
| WINP = Winter precipitation: (nov+dec+jan+feb), | 0.0002 |
| Mn_ppm_ = manganese concentration in the soil (ppm) | 0.0005 |
| K_CEC_ = proportion of potassium in CEC (cation exchange capacity) | 0.009 |
| GSP = Growing season precipitation (April to September) | -0.0003 |
| *Arbutus xalapensis =* frequency of occurrence of *Arbutus xalapensis* in the neighborhood | -0.085 |
| *Juniperus deppeana =* frequency of occurrence of *Juniperus deppeana* in the neighborhood | -0.047 |

**FIGURES**

**Supplementary Figure 1 |** Important variables for the model of incomplete seed based on a sample of 192 *Pinus strobifomis* trees and selected by ROC (Receiver Operating Characteristic) and the Random Forest algorithm. DD5 = Degree-days >5 degrees C (based on mean monthly temperature), GSP = Growing season precipitation (April to September), MMAX = Mean maximum temperature in the warmest month (degrees C), MTWM = Mean temperature in the warmest month (degrees C), MAP = Mean annual precipitation (mm), WINP = Winter precipitation: (nov+dec+jan+feb), GSDD5 = Degree-days >5 degrees C accumulating within the frost-free period, SPRP = Spring precipitation: (apr+may), SMRPB = Summer precipitation balance: (jul+aug+sep)/(apr+may+jun), Mn-ppm = manganese concentration in the soil (ppm), MMINDD0 = Degree-days <0 degrees C (based on mean minimum monthly temperature), SMRP = Summer precipitation: (jul+aug), MAT = Mean annual temperature (degrees C), dieback = *Cronartium ribicola* damage measurement in the crown, K-ppm = potassium concentration in the soil (ppm), Mg-CEC = proportion of magnesium in CEC (cation exchange capacity), K-CEC = proportion of potassium in CEC (cation exchange capacity), Pc *=* frequency of occurrence of *Pinus cooperi* in the neighborhood, %Om = Relative proportion of organic matter in CEC (%) in the soil, SMRSPRPB = ((Depreciated) Summer/Spring precipitation balance: (jul+aug)/(apr+may).

**Supplementary Figure 2 |** Important variables for the model of *Megastigmus albifrons* cone damage based on a sample of 192 *Pinus strobifomis* trees and selected by PLS (Partial Least Squares) and the Random Forest algorithm. %Om = Relative proportion of organic matter in CEC (%) in the soil, %Sat = percent of saturation, Zn-ppm = zinc concentration in the soil (ppm), K-CEC = proportion of potassium in CEC (cation exchange capacity), MTWM = Mean temperature in the warmest month (degrees C), Reg Ps = *Pinus strobiformis* regeneration, Cu-ppm = copper concentration in the soil (ppm), Mn-ppm = manganese concentration in the soil (ppm), HC = Hydraulic conductivity (cm/h), MMAX = Mean maximum temperature in the warmest month (degrees C), CEC = Cation exchange capacity, Fe-ppm = iron concentration in the soil (ppm), GSDD5 = Degree-days >5 degrees C accumulating within the frost-free period, SMRPB = Summer precipitation balance: (jul+aug+sep)/(apr+may+jun), MAP = Mean annual precipitation (mm), SDAY = Julian date of the last freezing date of spring, Mg-CEC = proportion of magnesium in CEC (cation exchange capacity), GSP = Growing season precipitation (April to September), Na-CEC = proportion of sodium in CEC (cation exchange capacity), Na-ppm = sodium concentration in the soil (ppm).

**Supplementary Figure 3 |** Important variables for the model of *Leptoglossus occidentalis* seed damage based on a sample of 192 *Pinus strobifomis* trees and selected by ROC (Receiver Operating Characteristic) and the Random Forest algorithm. SMRPB = Summer precipitation balance: (jul+aug+sep)/(apr+may+jun), DD5 = Degree-days >5 degrees C (based on mean monthly temperature), WINP = Winter precipitation: (nov+dec+jan+feb), Mn-ppm = manganese concentration in the soil (ppm), K-CEC = proportion of potassium in CEC (cation exchange capacity), GSDD5 = Degree-days >5 degrees C accumulating within the frost-free period, GSP = Growing season precipitation (April to September), MAP = Mean annual precipitation (mm), Ax = frequency of occurrence of *Arbutus xalapensis* in the neighborhood*,* Jd = frequency of occurrence of *Juniperus deppeana* in the neighborhood, MTWM = Mean temperature in the warmest month (degrees C), SMRP = Summer precipitation: (jul+aug), MMAX = Mean maximum temperature in the warmest month (degrees C), MMINDD0 = Degree-days <0 degrees C (based on mean minimum monthly temperature), dieback = *Cronartium ribicola* damage measurement in the crown, SPRP = Spring precipitation: (apr+may), Mg-CEC = proportion of magnesium in CEC (cation exchange capacity), SMRSPRPB = ((Depreciated) Summer/Spring precipitation balance: (jul+aug)/(apr+may), K-ppm = potassium concentration in the soil (ppm), Ca-ppm = calcium concentration in the soil (ppm).
